# BRAF^V600E^‐PROTAC versus inhibitors in melanoma cells: Deep transcriptomic characterization

**DOI:** 10.1002/ctm2.70251

**Published:** 2025-03-05

**Authors:** Solomon O. Alhassan, Zakaria Y. Abd Elmageed, Youssef Errami, Guangdi Wang, Joe A. Abi‐Rached, Emad Kandil, Mourad Zerfaoui

**Affiliations:** ^1^ Department of Gastrointestinal Oncology Moffitt Cancer Center Magnolia Campus Tampa Florida USA; ^2^ Department of Pharmacology, Edward Via College of Osteopathic Medicine University of Louisiana Monroe Louisiana USA; ^3^ Department of Microbiology Immunology and Molecular Genetics La Jolla California USA; ^4^ RCMI Cancer Research Center and Department of Chemistry Xavier University of Louisiana New Orleans Louisiana USA; ^5^ Tulane University School of Medicine New Orleans Louisiana USA; ^6^ Center for ViroScience and Cure, Department of Pediatrics, Laboratory of Biochemical Pharmacology Emory University School of Medicine Atlanta Georgia USA

**Keywords:** BRAF^V600E^, differentiation state, MAPK pathway, melanoma, MITF, PROTAC

## Abstract

**Aims:**

This study compares the suppression of Mitogen‐activated protein kinase (MAPK) signalling and early resistance potential between a proteolysis‐targeting chimera (PROTAC) and inhibitors targeting BRAF^V600E^.

**Methods:**

We performed a detailed in silico analysis of the transcriptomic landscape of the A375 melanoma cell line treated with a PROTAC and BRAF^V600E^ inhibitors from RNA sequencing data. The study assessed gene dysregulation, MAPK and Phosphoinositide‐3‐kinase (PI3K/AKT) pathway inhibition, and cell survival. Key genes uniquely dysregulated by PROTAC treatment were validated by qPCR. Furthermore, analysis was performed to evaluate dedifferentiation and early resistance signatures to understand melanoma drug‐induced plasticity.

**Results:**

PROTAC‐treated cells showed significantly lower MAPK pathway activity, strong cell cycle arrest and elevated apoptotic gene expression compared to inhibitor‐treated cells, with no effect on the PI3K/AKT pathway. A high microphtalmia‐associated transcription factor (MITF)/Tyrosine‐Protein Kinase Receptor (AXL) ratio in PROTAC‐treated cells indicated reduced early drug resistance. BRAF degradation induced a melanocytic‐transitory phenotype. Although PROTAC and inhibitor treatments caused overlapping transcriptomic changes, key differences were observed. PROTAC treatment enriched processes such as epithelial‒mesenchymal transition, inflammatory responses, and Tumor necrosis factor‐Alpha (TNF‐α) and IL2/STAT5 signalling.

**Conclusion:**

PROTAC‐targeting BRAF^V600E^ demonstrates enhanced MAPK suppression, reduced early resistance and distinct transcriptional effects compared to traditional inhibitors. It represents a promising strategy for overcoming resistance in melanoma treatment.

## Graphical abstract

Schematic depiction of MAPK signalling pathway and its cellular effects within the context of BRAF^V600E^ inhibition and PROTAC‐mediated degradation.

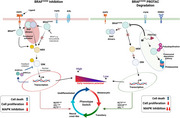

## 1 INTRODUCTION

The MAPK pathway is deregulated in melanoma, driven by mutations including NRAS, NF1, MEK1/2 and BRAF.^1^ BRAF^V600E^ mutation drive the constitutive hyperactivation of the MAPK pathway, which promotes cellular growth/proliferation and dedifferentiation of melanoma cells through alterations in the transcription and translations of many genes.^2^ Targeted inhibition of BRAF^V600E^ and MEK with dabrafenib, vemurafenib, encorafenib, trametinib, etc., leads to significantly improved clinical response, but adaptive resistance typically occurs within 12 months and is accompanied by risk of metastasis and death.^3^ Resistance to targeted inhibitors is accomplished through mechanisms that lead to the recovery of MAPK/ERK signalling or activation of compensatory/alternative pathways such as the PI3K/AKT pathway.^4^ These mechanisms include the overexpression of EGFR, ERBB3 and PDGFRβ, secondary BRAF mutations or amplification of BRAF, or use of alternative splice variants, etc. (reviewed in refs.^5^, ^6^). Although BRAF^V600E^ inhibitors are highly specific and effective in terminating the catalytic activity of the kinase, they are limited in preventing dimerisation of alternative RAFs (e.g., CRAF). This ‘draggability defect’ gives rise to paradoxical reactivation of MAPK even when targeted RAF kinases are inhibitor bound, and inhibitors are not present at saturating levels.^7^ Inhibitor‐induced co‐immunoprecipitation of ARAF, BRAF and CRAF has been reported, and induced dimerisation of cytoplasmic CRAF alone has been shown to trigger MEK phosphorylation.^8^ Indeed, the reactivation of the MAPK/ERK cascade is the primary outcome of resistance mechanisms in many cancers including melanomas (well‐reviewed in ref.^9^).

Therapies based on PROTACs have emerged as potentially better alternatives to kinase inhibitors. Such therapies combine the best properties of targeted inhibitors with the capacity of cells to effectively execute protein turnover and degradation.^10^ PROTACs are heterobifunctional molecules consisting of a ligand that interacts with a protein of interest, connected to a ligand recruiter of an E3 ubiquitin ligase via a linker molecule.^11^ The mechanisms of action of PROTACs distinguish them from classical small molecule inhibitors and offer several advantages, including positive clinical effects at lower dosages and frequencies. PROTAC treatment can lead to disease remission, reduced off‐target effects and efficacy even in the presence of drug‐resistance mechanisms.^12^, ^13^

A number of BRAF‐specific inhibitors, including PLX4032 (Vemurafenib), PLX8394 (plixorafenib), GSK2118436 (Dabrafenib) and BI‐882370^14^, ^15^ and can be used to design PROTACs of varying effectiveness. BI‐882370 and Encorafenib‐based BRAFV^600E^‐targeting PROTACs have been have shown the most in vitro degradation potential.^16^ In this study, we performed detailed gene expression and pathway‐centric enrichment analysis to evaluate the transcriptomic landscape of A375 cells treated with a BRAFV^600E^‐targeting PROTAC, its methylated analogue (which binds the oncogene but not the E3 ligase thus serving as a negative control for the PROTAC) and two BRAF^V600E^ inhibitors BI 882370 and vemurafenib. Our approach allows us to infer the effects on MAPK and PI3K/AKT activity, cell division, proliferation/quiescence states, the emergence of adaptive resistance and dedifferentiation/phenotype switching.

## 2 MATERIALS AND METHODS

### 2.1 Compounds

The PROTAC was designed with a ration approach and is composed three components—BI‐882370 as the BRAF^V600E^‐targeting warhead; pomalidomide to recruits the CUL4A E3 ubiquitin ligase cereblon (CRBN); and a flexible linker composed of four polyethylene glycol moieties. A 3.29 A X‐ray structure of the PROTAC bound to the BRAF kinase revealed an engagement of the ATB‐binding pocket of BRAF in the IN conformation of the DFG motif, a key structural switch regulating the shape and accessibility of the ATP‐binding pocket.^7^ A negative control for the PROTAC was designed by methylating the pomalidomide moiety within its glutarimide ring preventing interaction with the E3 ligase and will henceforth refer to as Me‐PROTAC throughout the manuscript. Cells were treated with these compounds as well as with negative controls (negative and Me‐PROTAC), BI‐882370 and vemurafenib.^7^

### 2.2 Data and methods

Uniformly processed count matrix of the dataset with accession number GEO: GSE148500^7^ was obtained from the Sequence Read Archive. These data are based on RNA sequencing of A375 melanoma cells treated at 200 nM for 20 h with the PROTAC and its methylated negative control directed at BRAF^V600E^. This cell line was also treated at the same concentration and for the same duration with vemurafenib and BI‐882370 (both BRAF^V600E^ inhibitors). Genes with counts less than 10 in 80% of the samples were filtered out and gene expressions were compared between PROTACs and inhibitors and to negative controls.

Normalisation (using the relative log expression method) and differential gene expression analysis were carried out using the Deseq2 package in R.^17^ Differentially expressed genes (DEGs) are defined as genes with false discovery rate‐adjusted *p*‐value <.05 and an absolute value of the log_2_ fold change ≥1.5 between comparisons.

Functional enrichment and pathway analysis were carried out using the web versions of Enrichr.^18^ Transcription factor enrichment was performed with CHEA3.^19^ Clustering of gene expression counts to identify co‐expressed genes was performed using the Clust method.^20^ The top 3000 genes with the most variance was selected (see Supporting Information S1 for cluster profiles and highly variable genes identified in the analysis). A principal component analysis (PCA) was performed on normalised count data using the FactoMineR^21^ R package. Gene set variation analysis was carried out using the *z*‐score method^22^ to compute pathway activity scores (a quantitative measure of the activity of specific pathways or cellular processes) for individual samples based on the expression of gene sets participating in a given pathway or process. Activity scores were computed using the following equation:

$$Z_{\gamma }=\frac{\sum_{i=1}^{n}z_{i}}{\sqrt{n}}$$

where *z_i_* is the *z*‐score of each gene's expression level, *n* is the number of genes in each gene set and $Z_{\gamma }$ is the combined *z*‐score for the gene set or a pathway activity score for the sample.

Heatmaps were constructed using the pheatmap^23^ package in R. Human cell cycle marker genes and their corresponding cycle stages were obtained from cyclebase 3.0,^24^ and *z*‐score normalised expression of genes were computed from normalised count data using the scale() function in base R. All analysis was carried out in R 4.1.3.

### 2.3 Validation by quantitative real‐time PCR

A375 melanoma cells treated with PROTAC SJF 0628 (Tocris Biosciences) at 200 nM for 20 h and negative controls. Total RNA was extracted from cells with the miRNeasy Micro Kit (Qiagen, cat no. 217084) and reverse transcribed to cDNA synthesised with SuperScript III First‐Strand Synthesis System (ThermoFisher Scientific, cat no. 18080051) according to manufactures recommendations. Quantitative real‐time PCR (qRT‐PCR) to validate genes of interest were performed with the Fast SYBR Green Master Mix (ThermoFisher Scientific, cat no. 4385612). The level of GAPDH served as an internal control and the relative expression was calculated based on the comparative Ct (2^−ΔΔCt^) method.^25^ See Supporting Information S2 for primer details.

## 3 RESULTS

### 3.1 Mechanism of PROTAC action on MAPK pathway and gene expression

**FIGURE 1:**
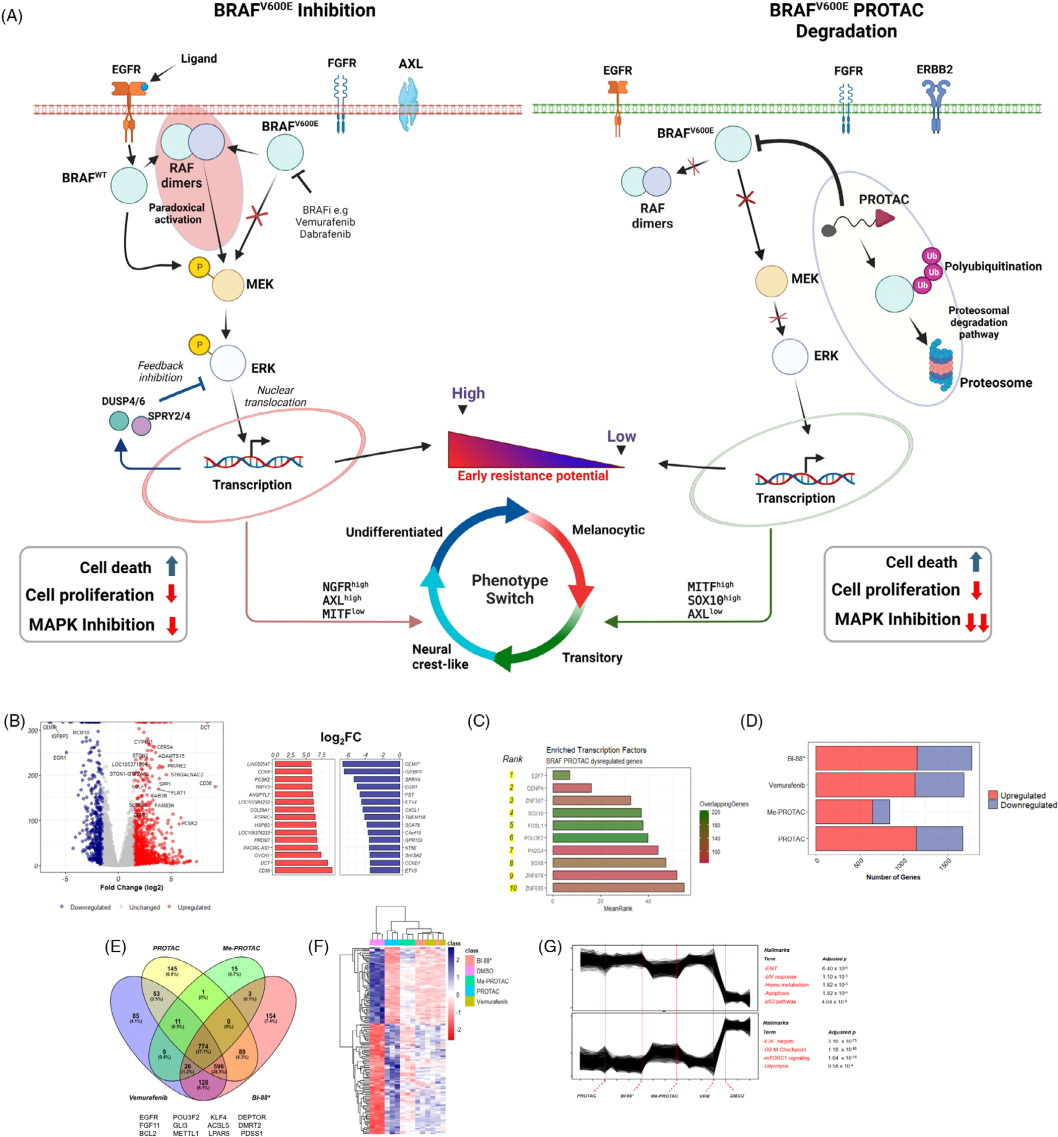
Transcriptional dysregulation with BRAF^V600E^ degradation or inhibition. (A) Schematic depiction of MAPK signalling pathway and its cellular effects within the context of BRAF^V600E^ inhibition and proteolysis‐targeting chimera (PROTAC)‐mediated degradation. (B) Volcano plot of differentially expressed genes in A375 cells treated with the PROTAC (right) and top 20 most differentially expressed genes (left) compared with Dimethyl sulfoxide (DMSO) controls. (C) Transcription factors significantly enriched in PROTAC‐treated cells. (D) Bar chart showing the number of dysregulated genes. (E) Venn diagram analysis comparing the significantly altered genes. Examples of genes dysregulated uniquely in PROTAC‐treated cells are listed under diagram. (F) Heatmap displaying normalised expression scores (*z*‐scores) of differentially expressed genes unique to PROTAC‐treated cells. (G) Clustering of genes based on expression of the top 3000 most variable genes in all samples. MSigDB hallmark pathway/process terms significantly enriched in each cluster is shown.

Inhibitors of BRAF^V600E^ suppress MAPK signalling and its downstream effects. However, the durability of cellular response induced by these compounds is hampered by induced expression of EGFR ligands and paradoxical activation of other RAF kinases with subsequent phosphorylation of MEK and reactivation of MAPK signalling (Figure 1C, left). This process also initiates adaptive resistance and elevated dedifferentiation of melanoma cells to more drug‐tolerant phenotypes. In contrast to inhibitors, PROTACs facilitate a proximity‐induced polyubiquitination BRAF^V600E^ via recruitment of E3 ubiquitin ligase complex. This not only achieves efficient blockage of MAPK signalling and output but has significantly less potential for inducing adaptive resistance leading to MAPK reactivation (Figure 1C, right).

Constitutive activation of the MAPK pathway through BRAF^V600E^ alters the transcriptional profile of cells. To understand the effects of BRAF^V600E^ degradation on the transcriptional landscape of A375 cells, we compared cells treated with the BRAF^V600E^‐PROTAC and negative controls and identified 529 downregulated and 1140 upregulated genes (Figure 1B). The most downregulated genes included well‐known targets of the MAPK pathway such as the transcription factors SOX11, MYC, EGR1 and E2F1; negative regulators DUSP2/4 and SPRY1/2/4; and growth and cell‐cycle regulators such as IGFBP3, HIPK and CDK2/6. With an enrichment analysis, the transcription factors E2F7, CENPA, SOX10, POU3F2 and FOSL1 were identified as significantly enriched in PROTAC‐treated cells (Figure 1C). To understand the transcriptomic effects of BRAF^V600E^ binding without the engagement of the ubiquitination machinery in the presence of linkers, we compared the expression levels of genes between the methylated analogue and negative controls and found 839 dysregulated genes (Figure S1A). BRAF^V600E^ inhibition by vemurafenib and BI‐882370 caused the differential expression of 1682 and 1770 genes, respectively (Figure 1D). We carried out a Venn diagram analysis to compare the landscape of altered genes in PROTAC or inhibitor‐treated cells and found a significant overlap with 774 genes common between the treatments (Figure 1E). This suggests a similar transcriptional response to interference with BRAF‐mediating signalling is induced either via inhibition or degradation, a notion supported by results from PCA analysis that showed a close clustering of PROTAC, or inhibitor‐treated groups (Figure S1B). This analysis also revealed 145 genes as differentially expressed uniquely with PROTAC, including well‐known signalling effectors such as EGFR and FGF11, transcription factors such as POU3F2 and KL4, and players in ferroptosis ACSL5 and LPAR5. These genes showed distinct patterns of expression (Figure 1F). When we compared cells treated with the PROTAC to inhibitor‐treated cells, only a small number of DEGs were identified, suggesting a partially similar response (Figure S1). Functional enrichment analysis of these shared genes revealed significant enrichment for terms such as ‘epithelial‒mesenchymal transition (EMT)’ (adj. *p*‐value = 7.48 × 10^−7^), ‘TNF‐α signalling via NF‐κB’ (adj. *p*‐value = 6.77 × 10^−4^), ‘IL2/STAT5 signalling’ (adj. *p*‐value = 4.54 × 10^−2^) and ‘hypoxia’ (adj. *p*‐value = 7.38 × 10^−2^) (Figure S1C).

Because genes are known to be expressed in co‐regulated clusters with distinct patterns of expression,^26^ we performed clustering analysis and enrichment analysis of differentially expressed by PROTAC treatment to identify their cellular function. Two tight clusters were identified: cluster 1 comprised genes enriched in processes such as EMT, cellular response to ultraviolet light, heme metabolism and the p53 pathway; cluster 2 genes were enriched in cell cycle‐related processes such as G2‐M checkpoint, mTORC1 signalling and glycolysis (Figure 1G). In summary, efficient degradation BRAF^V600E^‐induced significant transcriptional dysregulation with genes signatures comparable to its inhibition.

### 3.2 Pathway activation or inhibition and qPCR validation

**FIGURE 2:**
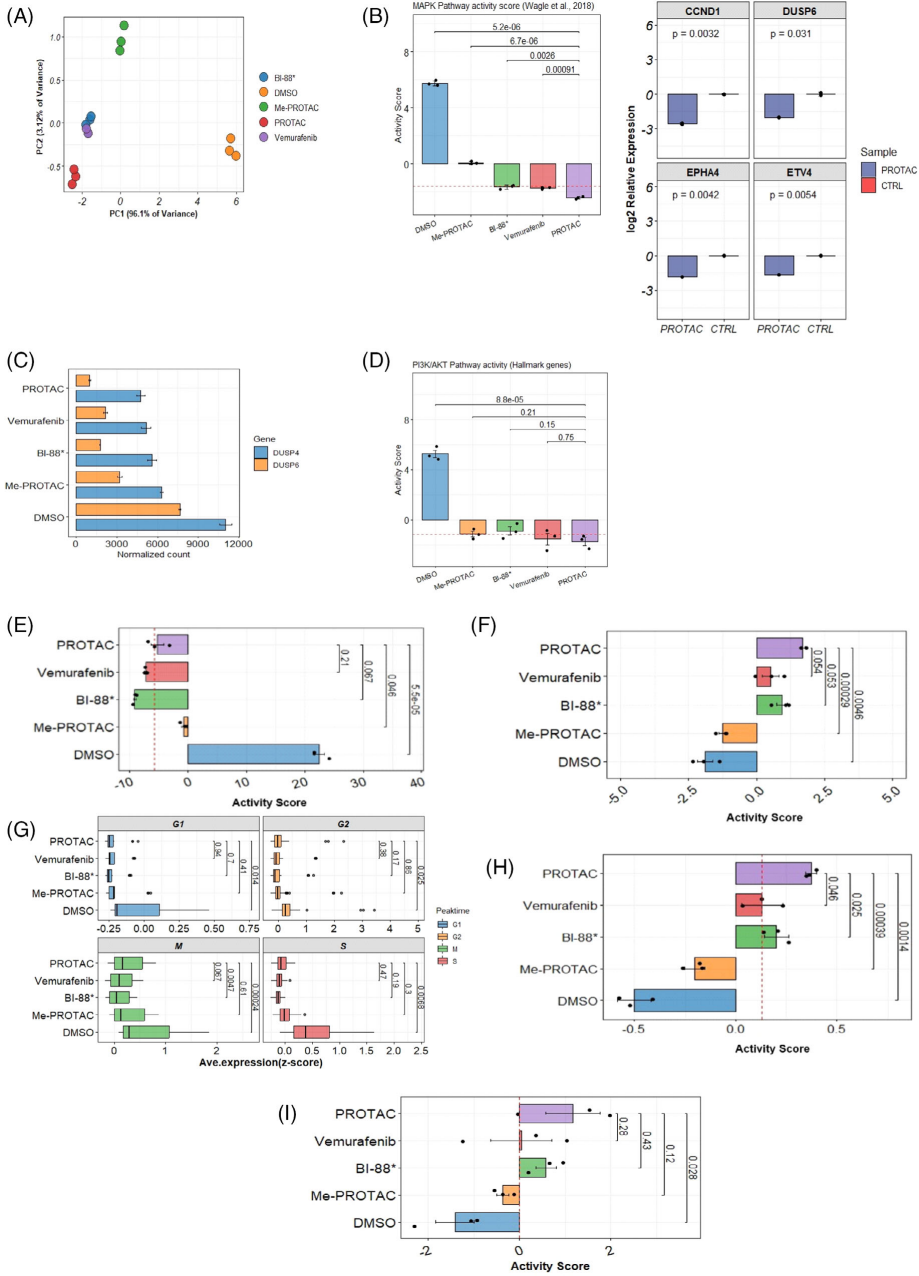
Pathway inhibition, cell proliferation and survival pathways induced by BRAF degradation. (A) Principal component analysis based on the expression of MAPK targets. Projections of the first two principal components (PC1‒PC2) are shown with percentage variance explained by each principal components indicated. (B, left) Pathway activity scores of clinically relevant downstream targets of the MAPK pathway in A375 cells treated with the proteolysis‐targeting chimera (PROTAC) or inhibitors. Gene set used in computing this score is derived from Wagle et al.^27^ (B, right) Quantitative polymerase chain reaction (qPCR) validation of four targets of the MAPK pathway in PROTAC SJF0628‐treated cells. (C) Expression levels of two dual‐specific phosphatase (DUSP) genes. (D) Pathway activity scores of MSigDB hallmark gene set for the PI3K/AKT pathway. (E) Cellular proliferation and (F) cellular quiescence. (G) Expression of cell cycle stage marker genes. Each cell cycle phase is indicated at the top of plot. Activity scores derived from expression of (H) pro‐apoptotic genes (I) mediators of ferroptosis. Negative scores indicate suppression of pathway/process. Red dashed line indicates cutoff derived from median of activity scores. Error bars: mean ± SE of biological replicates.

To assess the impact of BRAF^V600E^ degradation and inhibition on MAPK output and pathway‐level processes, pathway activity scores were computed for critical pathways downstream of or influenced by BRAF. First, we computed an activity score for the MAPK pathway for each treatment using a clinically validated gene set of conserved, downstream gene targets of the MAPK pathway^27^: SPRY2, SPRY4, DUSP4, DUSP6, CCND1, EPHA2, EPHA4, ETV4, ETV5 and PHLDA1. PCA showed that the expression of these 10 genes were able to explain 99.22% of the variability observed between treatment classes, suggesting that these genes are highly effective in distinguishing treatments by MAPK output (Figure 2A). A significantly lower pathway activity score (mean = ‒2.43, *p* = 5.2 × 10^−6^) was observed in PROTAC‐treated cells compared with negative controls (mean = 5.75), with the methylated‐PROTAC (mean = .067, *p* = 6.70 × 10^−5^), with BI‐882370 (mean = ‒1.66, *p* = 2.60 × 10^−3^), and importantly, even lower and significant (*p* = 9.10 × 10^−4^) than vemurafenib (mean = ‒1.73) (Figure 2B, left). We randomly selected four gene targets of MAPK signalling for qPCR validation by treating A375 cells with a comparable BRAF^V600E^‐PROTAC. We validated the significant downregulation of the key targets. CCND1 (log_2_ fold change = ‒2.55), DUSP6 (log_2_ fold change = ‒2.02), EPHA4 (log_2_ fold change = ‒1.85) and ETV4 (log_2_ fold change = ‒1.66) (Figure 2B, right). This result suggests that a more efficient blockade of MAPK signalling and consequent decrease in MAPK output can be achieved with BRAF degradation than with inhibition. A significant downregulation of the expression of several dual specific phosphatases (which fine‐tune or optimise MAPK signalling through ERK phosphorylation^28^) was observed in cells treated with the PROTAC (Figure 2C).

The existence of functional crosstalk between the MAPK and PI3K/AKT pathways in melanomas is well established.^4^ To understand the effects of PROTAC on the activity of this pathway, pathway activity scores were computed for the PI3K/AKT pathway using 88 genes from the AKT/PI3K/mTOR or MSigDB gene set (Figure 2D). A statistically significant (*p* = 8.8 × 10^−5^) lower activity of this pathway was observed in PROTAC‐treated cells (mean = ‒1.72) compared to negative controls (mean = 5.26). All other treated groups had lower PI3K/AKT activity scores than negative controls; however, no significant differences in the activity of this pathway were observed between PROTAC‐ and inhibitor‐treated cells. Our results thus suggest that BRAF^V600E^ degradation elicits a significantly more efficient blockage of MAPK signalling output and that of compensatory PI3K/AKT pathway than a just inhibition with important consequence to processes mediated by these pathways.

### 3.3 Cell proliferation and differentiation

To identify the effects of PROTAC and inhibition on cell proliferation at the transcriptomic level, we analysed the expression of genes for 157 markers of cellular proliferation.^29^ These include genes such as MKI67, MCM2‐7, PLK1, PCNA, etc. (Figure 2E). A significant positive correlation (*p* = 1.90 × 10^−10^, *r* = .98) between MAPK activity and proliferation was observed (Figure S1D). Cells treated with PROTAC had significantly lower proliferation scores compared with negative controls (mean ‒5.26 vs. 22.4, *p* = 5.5 × 10^−5^). However, no statistically significant difference in proliferation score was observed between PROTAC‐treated cells and cells treated with the inhibitors BI‐882370 (mean = ‒9.14) and vemurafenib (mean = ‒7.26). To complement this analysis, we evaluated the expression of markers of the G0, or cellular quiescence, state (CDKN1B/p27, CDKN1A/p21, NR2F1) to assess whether the treatments could trigger a reversible arrest in cell division (Figure 2F). A significantly higher activity score for these genes was observed in PROTAC‐treated cells compared with negative controls (mean = 1.70 vs. ‒1.89, *p* = 4.6 × 10^−3^) but not with cells exposed to vemurafenib or BI‐882370 though the effect of PROTAC treatment was higher in magnitude (Figure 2F). Furthermore, we profiled the expression of genes that mark each human cell cycle stage (Figure 2G). Compared to the negative controls, expression of cell cycle marker genes was significantly lower in PROTAC‐treated cells at the G1 (*p* = 1.40 × 10^−2^), G2 (*p* = 2.50 × 10^−2^), M (*p* = 2.4 × 10^−4^) and S (*p* = 6.80 × 10^−3^) phases. Similar expression levels at each cell cycle phase were observed between PROTAC‐ and inhibitor‐treated cells, except those in the M (mitotic) phase, which were significantly higher (*p* = 4.7 × 10^−3^) in PROTAC‐treated versus BI‐882370‐treated cells. Regarding the ability to induce cell death (Figure 2H, I), significantly higher activity of pro‐apoptotic genes (BCL2, BCL2L1, BAK1, MCL1, CASP3, CASP8, etc.) was observed in PROTAC‐treated cells compared with negative controls (mean = .37 versus ‒.51, *p* = 1.4 × 10^−3^) and vemurafenib‐treated (mean = .163, *p* = 4.6 × 10^−2^) and BI‐882370‐treated cells (mean = .20, *p* = 2.5 × 10^−2^) (Figure 2H). From a gene set composed of genes critically involved in ferroptosis, including GPX4, HMOX1, LPCAT5, ACSL5, TFRC, NOX1, NOX4 and HSPB1, we computed activity scores for each treatment class (Figure 2I). Cells treated with the PROTAC showed significantly higher activity for these genes than negative controls (mean = 1.17 vs. ‒1.42, *p* = 2.8 × 10); however, no differences were found between PROTAC‐ and inhibitor‐treated cells.

### 3.4 Dedifferentiation and resistance signatures

**FIGURE 3:**
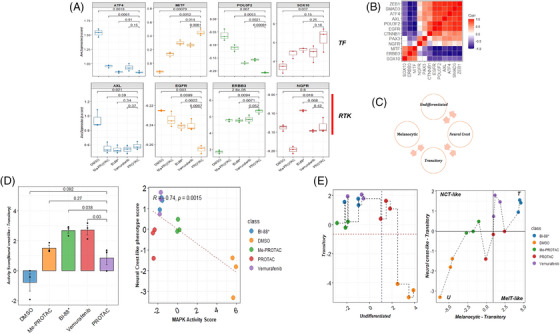
Profile differentiation subtype signature and dynamics with BRAF degradation. (A) Expression of select transcription factors and receptor tyrosine kinases that mediate differentiation state/phenotypes in melanomas. (B) Correlation in expression between key transcription factors and receptor tyrosine kinases acting with microphtalmia‐associated transcription factor (MITF) to determine differentiation state. (C) Schematic of melanoma differentiation trajectory. Adapted from Tsoi et al.^31^ (D) Neural crest‐like signature score for each treatment class (left) scatterplot showing relationship between MAPK pathway activity and treatment‐induced neural crest‐like phenotype (right). (E) Dedifferentiation position/stage of each treatment class. Differentiation scores are computed from expression of differentiation subtype signatures. Subtype signatures are obtained from Tsoi et al.^31^ Horizontal and vertical lines indicate cutoff derived from median of scores for each measure. MelT: melanocytic‐transitory; NCT‐like: neural crest‐like transitory; T: transitory; U: undifferentiated. Error bars: mean ± SE of biological replicates.

It is well known that targeted MAPK therapy induces phenotype switch and therapy resistance in melanoma cells through a highly complex interplay of transcription factors, receptor tyrosine kinases and epigenetic remodelers.^30^ To understand the effects of PROTAC on the early stages (within 24 h) of drug‐induced dedifferentiation in melanoma, we profiled the expression and activity of key transcription factors and receptor tyrosine kinases that effect a dedifferentiation program in treated cells (Figure 3A). These include the transcription factors microphtalmia‐associated transcription factor (MITF), SOX10, POU3F2/BRN2 and ATF4, and the receptor tyrosine kinase AXL, EGFR, ERBB3 and NGFR. A significant elevation of the melanocyte master transcription factor MITF was observed in cells treated with the PROTAC compared with other treatment groups and negative controls (*p* = 7.90 × 10^−4^). No differences in SOX10 and ATF4 expression were observed between cells treated with the PROTAC or inhibitors (Figure 3A, top). The expression levels of other key TFs, such as SMAD3 and ZEB1, were significantly higher in negative controls and BI‐882370‐treated cells than in PROTAC‐treated cells (Figure S2). However, the expression of the important tyrosine kinase AXL was significantly higher (*p* = 2.10 × 10^−2^) in negative controls than in PROTAC‐treated cells. No differences in expression levels of this gene were observed between PROTAC‐ and inhibitor‐treated cells. Interestingly, only in Me‐PROTAC‐treated cells was the expression of NGFR was significantly lower (*p* = 1.8 × 10^−2^) than in PROTAC‐treated cells, and EGFR was under‐expressed in BRAF‐degraded cells compared to negative control or inhibitor‐treated cells. The ERBB3 gene was significantly overexpressed in PROTAC‐treated compared to BI‐882370‐treated cells (Figure 3A, bottom). Correlation analysis revealed significant and strong positive correlations (*p* > .7) between MITF, SOX10 and ERBB3. Also, there were significant and strong negative correlations (*p* < ‒.7) between MITF and AXL, POUEF2, SMAD3 and EGFR (Figure 3B). To determine the differentiation state of cells in each treatment, gene set activity scores were computed using the expression of signatures that mark a differentiation subtype/stage (Figure 3C) in melanoma cells.^31^ Negative‐treated cells had the highest undifferentiated state scores, significantly higher (*p* = 2.8 × 10^−2^) than PROTAC‐treated cells. Me‐PROTAC‐, vemurafenib‐ and BI‐882370‐treated cells had negative undifferentiated state scores, which were all significantly lower (*p* < .05) than PROTAC‐treated cells (Figure S2B, left). All treated groups had positive transitory state scores, while negative controls had a (negative) transitory state score significantly lower than in PROTAC‐treated cells (*p* = 3.9 × 10^−4^) (Figure S2B, right). However, cells treated with Me‐PROTAC had a weakly positive transitory state score (mean = .011).

The emergence of a transient, neural crest‐like phenotype has previously been identified as an early‐stage adaptive response to MAPK inhibitors. This phenotype has also been linked to a reversible drug‐resistant state.^32^ Using expression of genes that mark this phenotype, we observed significantly higher neural crest‐like phenotype score in BI‐882370‐ and vemurafenib‐treated cells compared to PROTAC‐treated cells (Figure 3D, left). This strongly suggests that both inhibitor treatments induced a significant transition to a neural crest‐like phenotype than PROTAC treatment within 24 h of treatment. However, when PROTAC‐treated cells were compared with negative or Me‐PROTAC‐treated controls, no significant differences were observed (Figure 3D). Furthermore, a negative and significant correlation (*r* = .74, *p* = 1.5 × 10^−3^) between MAPK activity score and the neural crest‐like phenotype was observed (Figure 3D, right). This supports the evidence that MAPK pathway blockade achieved through BRAF inhibition/degradation drives the emergence of this phenotype. To more precisely identify the dedifferentiation position of cells based on gene expression signatures, we computed activity scores and traced differentiation state between treatment groups. Our results revealed an undifferentiated state in negative controls but a transitory phenotype state in PROTAC‐ and inhibitor‐treated cells (Figure 3E, left). Vemurafenib and BI‐882370‐treated cells showed the most neural crest‐like phenotype but with also strong expression of genes that define a melanocytic‐transitory state. PROTAC‐treated cells were inferred to be in a weakly melanocytic and neural crest‐like state that suggestive of weak inducement of a phenotype switch (Figure 3D, right).

**FIGURE 4:**
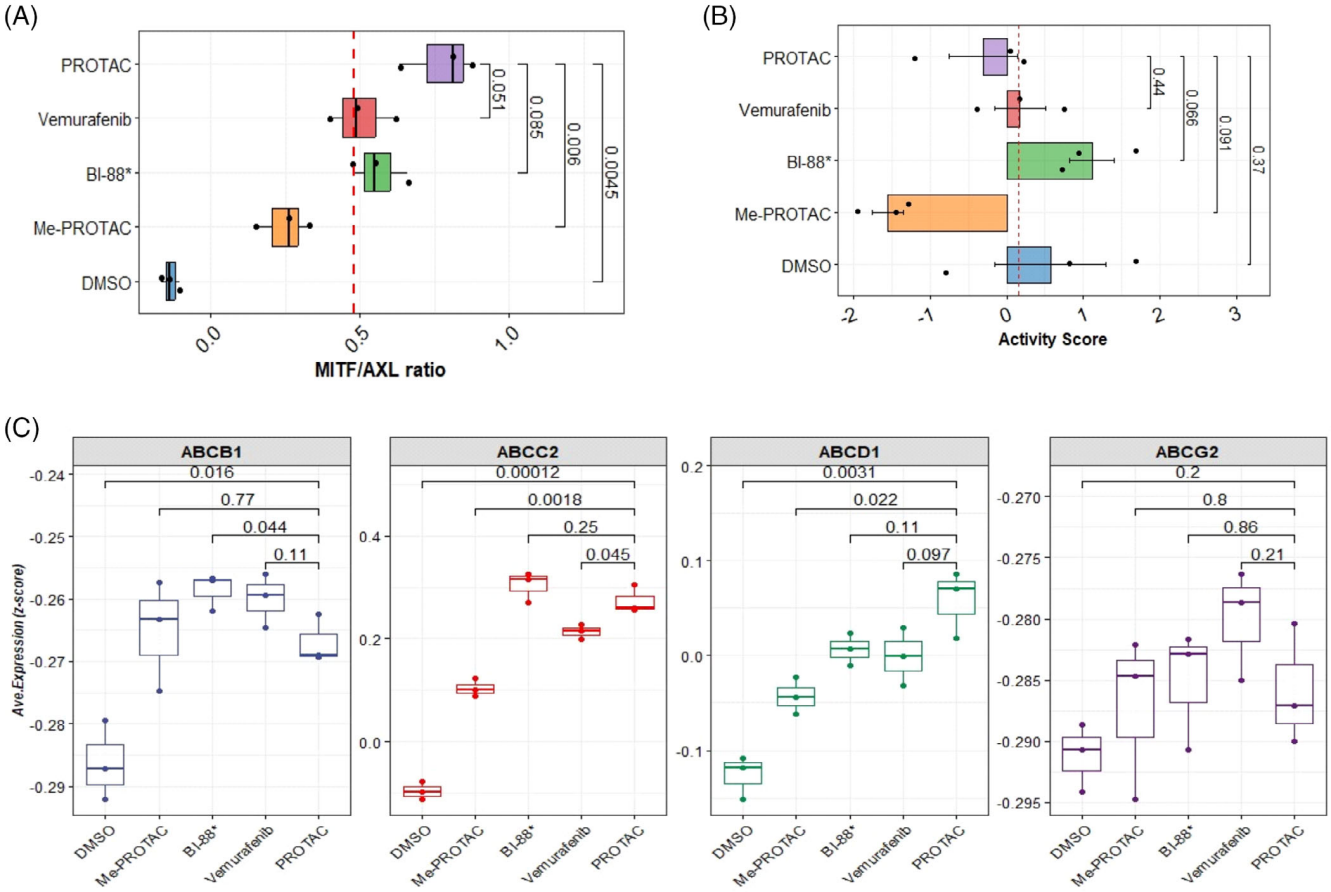
Early expression of therapy resistance signatures. (A) The ratio of microphtalmia‐associated transcription factor (MITF) to AXL receptor tyrosine kinase genes used as marker for the development of early resistance to multiple targeted therapies. (B) mRNA expression level of select genes that mark an intrinsic resistance phenotype in A375 cells. This gene set is derived from Zerfaoui et al. (2022). (C) Expression of genes for ABC family of drug efflux proteins. Horizontal and vertical lines indicate cutoff derived from median of scores for each measure. Error bars: mean ± SE of biological replicates.

Given the observed pattern of expression of key transcriptional drivers of dedifferentiation and phenotypic states associated with melanocyte development/plasticity, we interrogated the early markers of drug resistance in treated cells. A low MITF/AXL ratio predicts early resistance to multiple targeted drugs.^33^ To further determine whether an early resistance phenotype could be observed in treated cells, we calculated the MITF/AXL ratio. A high MITF/AXL ratio was observed in PROTAC‐treated cells compared with negative controls (*p* = 4.5 × 10^−3^) and Me‐PROTAC‐treated cells (*p* = 6.0 × 10^−3^). This ratio was higher, but not significantly so, in PROTAC‐treated cells compared with both inhibitors (Figure 4A). In our recent published work, the proteins HMOX‐1, BIRC5, CTSS, ENG, ICAM1, LGALS3, SPARC and DKK1 were identified as significantly upregulated in cells intrinsically resistant to BRAF^V600E^ inhibitors and are associated with a highly aggressive phenotype and relapse in patient samples.^34^ We computed resistance scores based on the expression of genes for these proteins for each treatment class (Figure 4B). No statistically significant difference in intrinsic resistance was observed between PROTAC‐treated cells and negative controls (*p* = .37) or when any other treatment groups is compared to PROTAC‐treated cells. The acquisition of resistance to targeted therapy, even to PROTACs, has been shown to result from increased expression and function of drug efflux pumps.^15^, ^35^ We profiled the expression of genes for several key ATP‐binding cassette (ABC) family transporters including ABCB1, which codes for the well‐known multidrug resistance 1 (MDR1) protein. The expression of ABCB1 was significantly lower (*p* = 4.4 × 10^−2^) in PROTAC‐treated cells compared to BI‐882370‐treated cells. However, no significant difference in ABCB1 expression could be observed between PROTAC‐ and vemurafenib‐treated cells. Expression was significantly higher for ABCC2 and ABCD1 (*p* < .01) in PROTAC‐treated cells compared with negative. There was no statistically significant difference in the expression of ABCG2 (a key mediator of resistance to vemurafenib) between PROTAC‐ and vemurafenib‐treated cells but the median expression of this gene was higher in magnitude in vemurafenib‐treated cells (Figure 4C).

### 3.5 Validation of PROTAC unique genes

**FIGURE 5:**
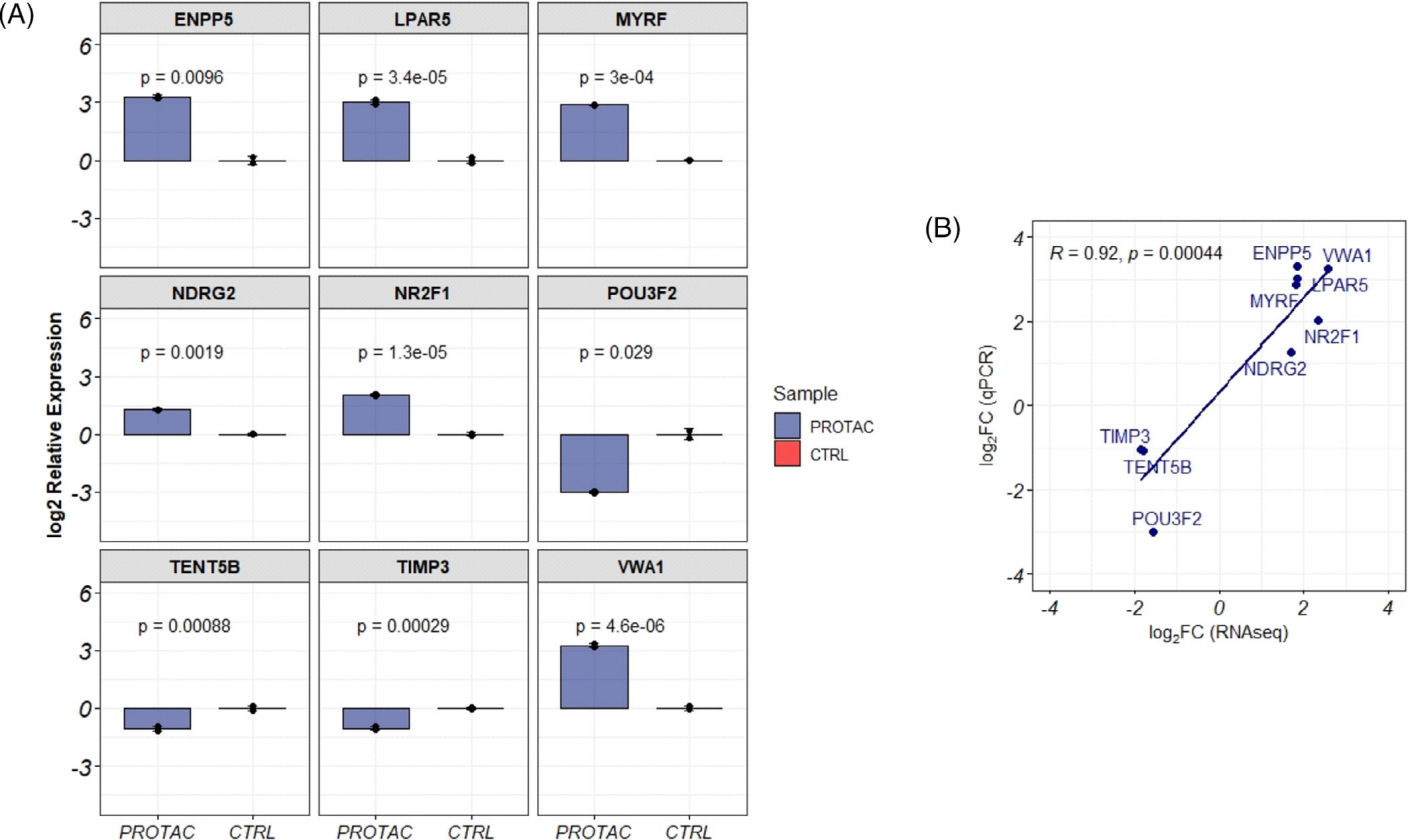
Validation of proteolysis‐targeting chimera (PROTAC) dysregulated genes and schematic model. (A) Top genes identified as dysregulated uniquely by PROTAC treatment were validated via quantitative polymerase chain reaction (qPCR) in A375 cells treated at 200 nM for 20 h with BRAF^V600E^‐targeting PROTAC SJF 0628. All assays were performed in triplicates. (B) Correlation between qPCR and RNA sequencing measurement for validated genes.

qRT‐PCR was used to validate the expression of genes identified as uniquely differentially expressed in PROTAC‐treated cells. Genes were selected for validation based on their fold change and function. In cells treated with PROTAC, we validated the significant downregulation of the POU3F2 (log_2_ fold change = ‒3.0, a key transcription factor regulating the expression of MITF), TIMP3 (log_2_ fold change = ‒1.03, an inhibitor of matrix metalloproteinase) and TENT5B (log_2_ fold change = ‒1.07, a gene involved in mRNA stabilisation). Furthermore, the expressions of ENPP5 (log_2_ fold change = 3.30), LPAR5 (log_2_ fold change = 2.87), MYRF (log_2_ fold change = 2.87), NDR2G (log_2_ fold change = 1.28) and NR2F1 (mean relative log_2_ fold change = 2.03) were validated as upregulated in PROTAC‐treated cells (Figure 5A). The fold change quantifications between qPCR and RNAseq measurement of these genes were significantly correlated (*R* = .92, *p* = 4.4 × 10^‒4^) (Figure 5B).

## 4 DISCUSSION AND CONCLUSION

Treatment for melanomas depends largely on BRAF mutational status and the use of targeted inhibitors. Acquired drug resistance to inhibitor therapy is rapid, necessitating the development of novel modalities such as PROTACs, which target and degrade proteins by leveraging the ubiquitin proteosomal system.^11^

In this study, we observed that BRAF^V600E^ degradation using a PROTAC induces a strong transcriptional response with the dysregulation of many important genes and to an extent comparable in magnitude to BRAF^V600E^ inhibitors. Notably 73.7% of genes dysregulated by PROTAC treatment (compared to untreated controls) overlap with those dysregulated in BI 882370‐treated cells likely because both achieve an overall similar outcome (the arrest of the MAPK pathway) and utilise a similar BRAF^V600E^ engagement mechanism (binding of the ATP‐binding pocket) although the PROTAC even goes further by recruiting the E3 ligase. Importantly, PROTACs based on BI 882370 have been shown to be more effective at BRAF^V600E^ degradation than those based on the structurally different but also potent inhibitor dabrafenib.^7^ This also underscores the importance of structural and biophysical/interaction considerations in the design of effective PROTACs for melanoma therapy.^36^

We leveraged the expression of 10 of these genes highly influence by MAPK signalling and validated to highly predictive of response to MAPK inhibitors in both clinical studies and multiple cell lines to quantify the degree of MAPK suppression achieved by BRAF^V600E^ degradation and compared this to effects achieved by inhibitors. Our results show that efficient BRAF^V600E^ degradation achieves a significantly better downgrade of this oncogenic pathway. This is likely because degradation removes the BRAF^V600E^ from cells, whereas inhibitors block catalytic functions, but not necessarily other non‐enzymatic functions such as the BRAF‐induced activation of other RAFs. Additional studies have shown that protein removal achieves a more pronounced cellular effect than inhibition; for example, degradation of EGFR, FLT‐3 and BRD4 achieved a more durable blockade of their respective pathways than inhibition, with a significant attendant decrease in cell growth.^37^ We also quantified the activity of the compensatory PI3K/AKT pathway and identified a similar magnitude of this suppression between PROTACs and inhibitors. This suggests that for acute responses where the biological importance of compensatory or alternative pathways may not be important, degradation may not hold strong advantages over inhibition. Additional studies are required to study cases in which more long‐term effects, such as the adaptive acquisition of resistance to inhibitors, are of interest.

Blunting ERK activation with inhibitors of upstream mediators of the pathway has been demonstrated in both clinical and in vitro studies to arrest cell growth and proliferation and initiate anti‐survival mechanisms.^38^ By profiling critical markers of proliferation, PROTAC treatment achieved the arrest of cell proliferation at levels comparable to inhibitors, but triggered apoptosis more than inhibitor‐treated cells. Furthermore, newer treatments such as PROTACs can induce vulnerabilities to other modalities of cell death such as ferroptosis in melanoma,^39^ although we did not observe a significant increase in ferroptosis signatures possibly due to the short exposure period.

The capacity of melanomas to switch states in the differentiation spectrum (from highly differentiated/melanocytic stage to undifferentiated states) is linked to the embryonic/neural crest origin of melanocytes. Our results point to a highly variable response to PROTAC or inhibition in the expression of a key lineage‐specific transcription factor, MITF, and the RTKs (AXL, ERBB2, EGFR and NGFR). We found MITF is significantly overexpressed in BRAF‐degraded cells compared with both inhibitor‐treated and control cells, and the expression of AXL, which is impacted by MITF levels or function,^40^ was significantly under‐expressed in PROTAC‐treated cells. This is consistent with results from studies showing that BRAF inhibition induces MITF^high^/AXL^low^ gene expression state in both human and in vitro studies.^41^ The elevated MITF expression in PROTAC‐treated cells corresponded with an under‐expression of the transcription factor POU3F2/BRN, a transcriptional repressor of MITF,^42^ and the overexpression of SOX10, with activates MITF expression,^43^ in the same cells. MITF is considered a driver of an early non‐mutation and reversible drug‐tolerant state induced by PAX3‐mediated upregulation of MITF.^42^ We also found a downregulation of EGFR and upregulation of ERBB3 in cells with degraded BRAF, a well‐established pattern with MAPK inhibition.^44^ These expression patterns are consistent with features of a melanocytic‐transitory state in PROTAC‐treated cells^31^ and suggest that PROTAC treatment is less likely to trigger early induction of resistance mechanisms than inhibitors. A surprising observation in these data is the existence of the untreated/negative A375 cells in MITF^low^/AXL^high^/SOX10^low^ expression state highly characteristic of undifferentiated cells. This state is even more confirmed by a SMAD3^high^/ZEB1^high^/EGFR^high^ expression pattern in this negative controls. This undifferentiated state gene expression pattern and a MITF^low^/AXL^high^ is associated with lack of sensitivity (or the presence of resistance) to single and/or double treatment with MAPK inhibitors.^31^ Several questions arise from this observation: Could negative induce an undifferentiated state in BRAF mutant A375 cells? Is differentiation status a flexible, dynamic state even without drug pressure in BRAF mutant cells? An affirmative answer to the latter question seems most likely. Bai et al.^45^ assert that cell‐state dynamics in melanomas are extremely complex and can be independent of the activity or expression of several genes regardless of their functional importance or hierarchy. As we inferred from transcriptomic profile of treated cells, an acute response in inhibitor‐treated cells was a rapid transition/de‐differentiation along the melanocyte lineage to a more neural crest‐like phenotype associated with drug tolerance. This highly suggests that BRAF^V600E^‐PROTACs are less likely to induce an adaptive resistance than inhibitors and are more clinically advantageous than inhibition. However, the characterisation of a more long‐term response is needed to verify this.

In conclusion, this study provides compelling evidence that the targeted degradation of BRAF^V600E^ via a PROTAC‐based approach achieves a more durable and comprehensive blockade of the MAPK signalling cascade than conventional BRAF^V600E^ inhibitors. By facilitating the proteasomal removal of BRAF^V600E^ rather than merely inhibiting its catalytic function, PROTACs prevent the protein‐level mechanisms that often lead to adaptive resistance, including paradoxical MAPK reactivation and compensatory pathway upregulation. This degradation‐driven strategy not only lowers the risk of cells shifting towards drug‐tolerant phenotypes but also maintains stronger and more persistent suppression of tumor growth and survival pathways.

From a clinical standpoint, these findings underscore the potential of PROTACs to offer a more sustained and robust therapeutic response in melanoma patients harboring BRAF^V600E^ mutations. Such a strategy could diminish the frequency and severity of acquired resistance observed with current therapies, ultimately extending the duration of clinical benefit, and improving patient outcomes. By harnessing the cell's intrinsic protein turnover mechanisms, PROTACs may therefore represent the next generation of precision oncology drugs, offering a superior and more durable alternative to conventional kinase inhibitors.

### 4.1 Limitations

This study focus on early, within 24‐h responses may not fully capture longer‐term dynamics of BRAF^V600E^ degradation and the development of resistance. However, the early time point provides critical insights into the immediate transcriptional responses which is essential to understanding early drug action. Although one cell line was used which might limits generalisability to other melanoma subtypes, A375 cell lines are a standard and well characterised model of BRAF^V600E^‐mutant melanoma, allowing a meaningful baseline assessment of the PROTAC's effect. Last, we understand that transcriptional changes alone may not reflect protein‐level outcomes; however, the study's integrative approach—combining transcriptomics with pathway analyses—provides a robust conceptual framework that can inform functional level outcomes and future in vivo and long‐term clinical studies.

## AUTHOR CONTRIBUTIONS

*Study conception and design*: Mourad Zerfaoui, Solomon O. Alhassan and Zakaria Y. Abd Elmageed. *Acquisition of data*: Solomon O. Alhassan. *Analysis and interpretation of data*: Solomon O. Alhassan, Mourad Zerfaoui and Youssef Errami. *Drafting of manuscript*: Solomon O. Alhassan and Mourad Zerfaoui. *Critical revision*: Mourad Zerfaoui, Solomon O. Alhassan, Zakaria Y. Abd Elmageed, Youssef Errami, Joe A. Abi‐Rached, Guangdi Wang and Emad Kandil.

## CONFLICT OF INTEREST STATEMENT

The authors declare they have no conflicts of interest.

## ETHICS Statement

The authors have nothing to report.

## Supporting information



Supporting Information

**TABLE S1**. Cluster profiles and highly variable genes identified in the analysis.

**TABLE S2**. Primer sequences and details used in the study.

## Data Availability

The data that support the findings of this study are available from the corresponding author upon reasonable request.
