# Supplementary material for: BRAFV600E‐PROTAC versus inhibitors in melanoma cells: Deep transcriptomic characterization
Source: Clin Transl Med. 2025 Mar 5;15(3):e70251. doi: 10.1002/ctm2.70251 (PMC11882472; doi:10.1002/ctm2.70251)
Supplement: Supplementary file 1 — Supporting Information [file CTM2-15-e70251-s002.pdf]

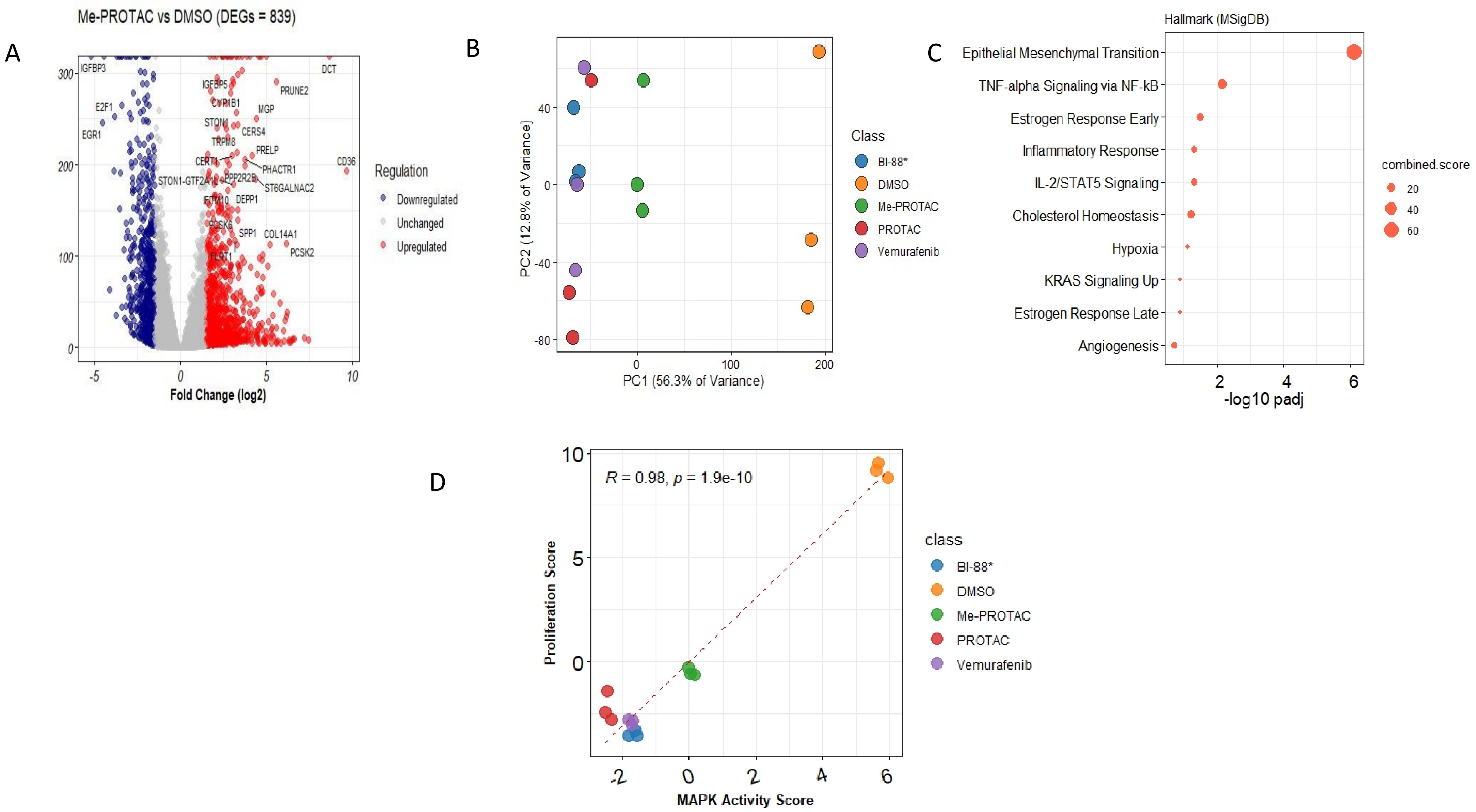
A
Me-PROTAC vs DMSO (DEGs = 839)
Fold Change (log2)
Regulation
Downregulated
Unchanged
Upregulated
IGFBP3
E2F1
EGR1
DCT
PRUNE2
MGP
CERS4
PRELP
PHACTR1
CD36
ST6GALNAC2
DEPP1
SPP1
COL14A1
PCSK2
B
PC1 (56.3% of Variance)
PC2 (12.8% of Variance)
Class
BI-88*
DMSO
Me-PROTAC
PROTAC
Vemurafenib
C
Hallmark (MSigDB)
Epithelial Mesenchymal Transition
TNF-alpha Signaling via NF-kB
Estrogen Response Early
Inflammatory Response
IL-2/STAT5 Signaling
Cholesterol Homeostasis
Hypoxia
KRAS Signaling Up
Estrogen Response Late
Angiogenesis
-log10 padj
combined.score
D
R = 0.98, p = 1.9e-10
Proliferation Score
MAPK Activity Score
class
BI-88*
DMSO
Me-PROTAC
PROTAC
Vemurafenib

Figure S1: (A) Differentially expressed genes in A375 cell treated with methylated analog of PROTAC. **(B)** Principal Component analysis of A375 cells treated with PROTAC and inhibitors. Projections of the first two principal components (PC1 and PC2) are shown with percentage of variances explained by each is indicated. **(C)** Significantly enriched (adjusted $p < 0.05$) gene ontology (Hallmark) terms genes differentially expressed common to both inhibitors and PROTAC **(D)** Scatterplot of proliferation scores and MAPK activity scores.

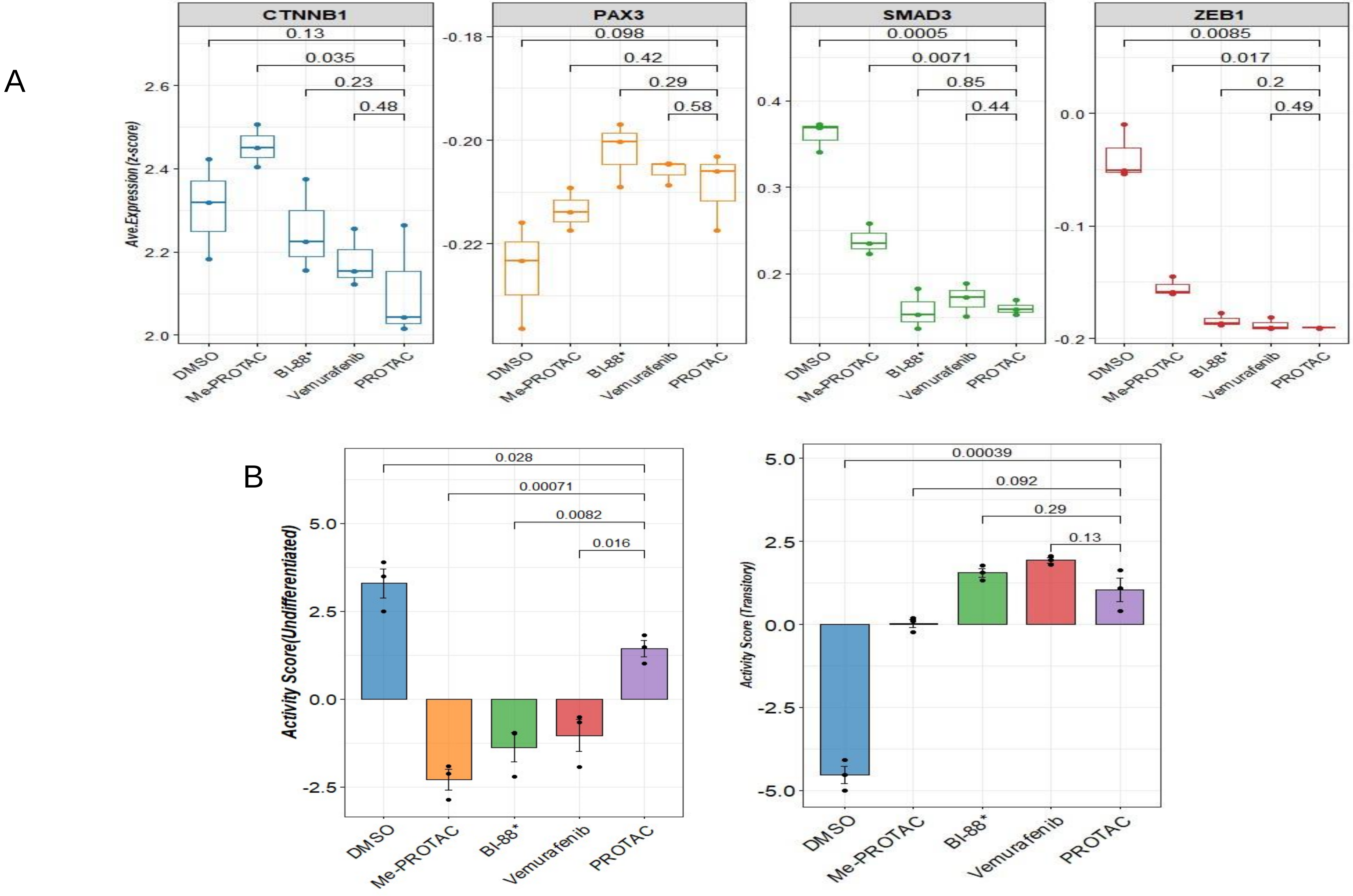
A
CTNNB1
PAX3
SMAD3
ZEB1
Ave.Expression (z-score)
0.13
0.035
0.23
0.48
0.098
0.42
0.29
0.58
0.0005
0.0071
0.85
0.44
0.0085
0.017
0.2
0.49
2.6
2.4
2.2
2.0
-0.18
-0.20
-0.22
0.4
0.3
0.2
0.0
-0.1
-0.2
DMSO
Me-PROTAC
BI-88*
Vemurafenib
PROTAC
B
Activity Score(Undifferentiated)
0.028
0.00071
0.0082
0.016
5.0
2.5
0.0
-2.5
Activity Score (Transitory)
0.00039
0.092
0.29
0.13
5.0
2.5
0.0
-2.5
-5.0
DMSO
Me-PROTAC
BI-88*
Vemurafenib
PROTAC

Figure S2: (A) Average expression of key transcription factors effecting a differentiation program in melanoma cells. Expression is derived from z-score normalized value of each gene. **(B)** Activity of genes defining an undifferentiated and transitory state phenotype in melanoma in PROTAC, and inhibitor treated cells. Subtype signatures are obtained from Tsoi et al., 2018. Error bars: Mean ± SE of biological replicates
